# T cell landscape within primary melanoma as a biomarker of survival after cancer vaccination in patients with metastatic disease

**DOI:** 10.1186/2051-1426-3-S2-P420

**Published:** 2015-11-04

**Authors:** Angela Vasaturo, Altuna Halilovic, Kalijn Bol, Dagmar Verweij, Willeke Blokx, Patricia Groenen, Han van Krieken, Johannes Textor, Jolanda de Vries, Carl Figdor

**Affiliations:** 1 RIMLS, Nijmegen, Netherlands; 2 RadboudUMC, Nijmegen, Netherlands; 3 RIMLS, Radboudumc, Nijmegen, Netherlands

## 

Melanoma is a highly malignant melanocyte-derived tumor and its incidence is increasing at outstanding rate. Despite different types of immunotherapy became available for the treatment of melanoma, including adoptive T cell transfer, immune checkpoint blockade, and vaccines such as dendritic cell vaccines, when applied to treat metastatic melanoma, only 10 to 40% of the patients have long term benefit. Furthermore, given the toxicity and high costs associated with immunotherapy there is a stringent need to identify biomarkers that may predict its potential efficacy. We have investigated whether the presence and distribution of T cells within the primary tumor of melanoma patients correlates with survival when treated for metastatic disease with dendritic cell based cancer vaccines. Quantitative multispectral imaging has been used to compare the tumor microenvironment of responding patients that survived longer than 20 months after immunotherapy, and non-responding patients that survived less than 12 months after immunotherapy. T cell infiltrates have been initially assessed in primary melanomas of 19 metastatic melanoma patients treated with dendritic cell based immunotherapy (discovery set) and subsequently in an independent cohort of 39 patients (validation cohort). In the discovery cohort we observed a very high correlation between the ratio of peritumoral over intratumoral T cells in the primary tumor and overall survival. Lower peri/intratumoral T cell ratios in primary tumors were associated with improved clinical outcome. ROC curve and multivariate analysis were exploited to evaluate the predictive power of the T cell ratio and established prognostic markers. Statistical analyses in the validation cohort confirmed our findings showing that the peri/intratumoral T cell ratio correctly predicted long term survival after DC vaccination in 90% of the cases and was the strongest predictor of survival in the multivariate analysis. This study indicates that the ratio between CD3 positive peri/intra-tumoral T cells is a very strong predictor of patient outcome and response to dendritic cells immunotherapy in melanoma patients.

